# Patient and public involvement in randomised clinical trials: a mixed-methods study of a clinical trials unit to identify good practice, barriers and facilitators

**DOI:** 10.1186/s13063-021-05701-y

**Published:** 2021-10-23

**Authors:** Lucy Ellen Selman, Clare Clement, Margaret Douglas, Keith Douglas, Jodi Taylor, Chris Metcalfe, J. Athene Lane, Jeremy Horwood

**Affiliations:** Bristol Randomised Trials Collaboration, Bristol Trials Centre, University of Bristol, Canynge Hall, 39 Whatley Road, Bristol, BS8 2PS UK

**Keywords:** Qualitative research, Cross-sectional survey, Public involvement, Patient involvement, Randomised trials, Trial oversight, User involvement

## Abstract

**Background:**

While patient and public involvement (PPI) in clinical trials is beneficial and mandated by some funders, formal guidance on how to implement PPI is limited and challenges have been reported. We aimed to investigate how PPI is approached within a UK Clinical Trials Unit (CTU)’s portfolio of randomised controlled trials, perceived barriers to/facilitators of its successful implementation, and perspectives on the CTU’s role in PPI.

**Methods:**

A mixed-methods study design, involving (1) an online survey of 26 trial managers (TMs) and (2) Interviews with Trial Management Group members and public contributors from 8 case-study trials. Quantitative survey data were summarised using descriptive statistics and interview transcripts analysed thematically. Two public contributors advised throughout and are co-authors.

**Results:**

(1) 21 TMs completed the survey; (2) 19 in-depth interviews were conducted with public contributors (*n*=8), TMs (*n*=5), chief investigators (*n*=3), PPI coordinators (*n*=2) and a researcher. 15/21 TMs surveyed reported that a public contributor was on the trial team, and 5 used another PPI method. 12/21 TMs reported that public contributors were paid (range £10–50/h). 5 TMs reported that training was provided for public contributors and few staff members had received any formal PPI training. The most commonly reported tasks undertaken by public contributors were the review of participant-facing materials/study documents and advising on recruitment/retention strategies. Public contributors wanted and valued feedback on changes made due to their input, but it was not always provided. Barriers to successful PPI included recruitment challenges, group dynamics, maintaining professional boundaries, negative attitudes to PPI amongst some researchers, a lack of continuity of trial staff, and the academic environment. Successful PPI required early and explicit planning, sharing of power and ownership of the trial with public contributors, building and maintaining relationships, and joint understanding and clarity about expectations/roles. CTUs have an important role to play in supporting recruitment, signposting and coordinating PPI.

**Conclusions:**

While highly valuable, PPI in trials is currently variable. PPI representatives are recruited informally, may not be provided with any training and are paid inconsistently across trials. Study findings can help optimise PPI in trials and ensure researchers and public contributors are adequately supported.

**Supplementary Information:**

The online version contains supplementary material available at 10.1186/s13063-021-05701-y.

## Background

Patient and Public Involvement (PPI) in research is described as research being carried out ‘with’ or ‘by’ patients and members of the public rather than ‘to’, ‘about’ or ‘for’ them [1]. The importance of PPI in health research has for many years now been recognised and advocated [2]. There are clear ethical, societal and scientific reasons for PPI in research, and evidence of the beneficial impact of PPI on the quality and delivery of clinical research is mounting [3–5]. In particular, incorporating the perspectives of public contributors in research can improve its relevance and reduce research “waste” [6, 7]. As a result, PPI has become central to the strategic and operational development of research and health policy [8]. Some funding bodies, including the National Institute for Health Research (NIHR) in England, now require all applicants to actively include PPI in their research proposals and budgets, to improve the relevance, accountability and quality of research [9].

However, researchers have sought increased clarity about funders’ expectations regarding PPI and guidance on its delivery [8, 10, 11]. The NIHR organisation INVOLVE produced guidance on PPI for researchers in 2012 [12], but ongoing problems with implementing PPI have been reported. A systematic review [13] exploring the impact of PPI on public contributors, researchers and communities involved in health and social care research reports specific challenges: lack of preparation and training led some public contributors to feel unable to contribute to the research, while other public contributors and communities reported feeling overburdened with the work involved; researchers reported difficulties in incorporating PPI in meaningful ways due to lack of money and time. These findings highlight the importance of optimising the context and processes of involvement, to create the potential for PPI to impact positively on research.

PPI is thought to be of particular value in clinical trials [14], for example in shaping trial design and the selection of outcomes relevant to patients and improving recruitment [15, 16]. INVOLVE published additional notes on PPI for researchers working in trials [17], and a toolkit for PPI involvement in trials has been developed [18]. However, formal guidance about how to involve public contributors operationally in the conduct of trials is lacking, and problems implementing meaningful PPI which is not tokenistic have been reported [4, 19]. A modified Delphi process has highlighted key uncertainties about PPI in trials including understanding relationships between researchers and public contributors, exploring PPI practices and resource implications for PPI in trials [20]. While the evidence base to inform the implementation of PPI in trials remains limited, qualitative research has highlighted the need to carefully consider models for implementing PPI and reflect on facilitators of and barriers to success [19, 21–25].

To contribute to the evidence base and identify good practice, we aimed to investigate how PPI is approached within a UK Clinical Trial Unit (CTU)’s portfolio of randomised controlled trials (RCTs), identify barriers to and facilitators of its successful implementation and explore perspectives on the role of the CTU in PPI.

## Methods

A sequential explanatory mixed-methods design was used, in order to both determine how PPI was approached across eligible portfolio trials and explore barriers, facilitators and experiences of PPI within selected trials in more depth. The study is reported in line with Good Reporting of a Mixed-Methods Study (GRAMMS) quality guidance [26]. Initially, an online survey was conducted of trial managers of all current and recent trials (ending in the previous two years) on the portfolio of an UKCRC-registered CTU. Of these RCTs, 8 were selected as case studies and semi-structured qualitative interviews conducted with trial management group members and PPI contributors (henceforth ‘public contributors’). All data was collected in 2016.

### Setting and context: A CTU at a UK university

At the time of the study (2016), there was no CTU-coordinated PPI support or PPI training. This study was conducted in part to inform the development and improvement of PPI support at the CTU. Changes to practice over the last 5 years are described in the ‘Discussion’ section.

### Sampling and recruitment

#### Online survey

All current trials on the CTU portfolio and recent trials whose funding period ended in the previous 2 years were identified via the CTU database. Trial managers for each trial were identified and invited to complete the survey, which was not anonymous. Where the trial manager had left the employment of the university, the trial’s Chief Investigator (CI) was invited to complete the survey. To raise awareness of the study, the rationale, purpose and methodology of the study were introduced to trial managers at a CTU meeting prior to the study’s start.

#### Qualitative interviews

Eight case study trials were selected purposively to represent a range of trial designs, funding streams, trial initiation dates and responses to the survey. Four were feasibility/pilot trials and four were phase III RCTs. For each case study trial, the CTU Research Manager (JT) emailed the trial manager and/or CI, attaching a participant information sheet and asking them to contact the researchers if they were willing to be interviewed. The Research Manager also asked them to forward the information sheet to 1–2 public contributors from the trial, asking whether they would be happy for their contact details to be passed on to the research team. The research team then contacted trial staff and public contributors interested in taking part in the interviews to explain the study, answer any questions and, should they consent, arrange an interview. Snowball sampling was used to identify any additional trial staff who would be useful informants on PPI in the trial, with purposive sampling also used to ensure a diverse range of perspectives (TMs, CIs, trial PPI co-ordinators (where present), researchers). The sample size was informed by the concept of ‘information power’ [27], with analysis and sampling conducted in parallel and continuous assessment of the suitability of the information within the sample with regard to study objectives.

### Data collection

#### Online survey

Trial managers were invited to complete an online survey (Additional file 1) via an email from the CTU Research Manager. The survey questionnaire was developed by the study team based on the literature and study aims and piloted prior to dissemination. Two reminder emails were sent to non-responders within a 4-week period. The survey was administered via Online Surveys (https://www.onlinesurveys.ac.uk/).

#### Qualitative interviews

Interviews were conducted face-to-face or by telephone, depending on participant availability and preference, by an experienced qualitative interviewer with a background in clinical trials research (LS, JH or CC). To ensure that the primary issues were covered across all interviews, semi-structured topic guides were used, informed by the study aims, research team expertise and the literature (Additional files 2 and 3). Two public contributors (MD, KD) who had worked on several trials provided feedback on the draft topic guides and helped refine them prior to use. Topic guides were flexible to enable participants to introduce new issues unanticipated by the researchers and were modified iteratively during data collection to reflect emerging findings. With informed consent, interviews were digitally recorded, professionally transcribed and anonymised to protect confidentiality prior to analysis.

### Analysis

Survey and interview data were analysed separately before being combined in an integrated narrative. In accordance with an explanatory design, the qualitative data were used to enrich and develop quantitative findings.

#### Online survey

Quantitative data were downloaded into Excel and SPSS for descriptive analysis (LS). Qualitative free text related to public contributors’ background and ways in which the CTU could support PPI in trials were analysed using thematic content analysis [28, 29] (LS). Responses were categorised, tabulated and summarised narratively, with some quotes utilised in the results.

#### Qualitative interviews

Data were analysed thematically [30] in NVivo v10 [31]. LS constructed a draft coding frame using a combination of inductive coding [32], staying close to the data, and deductive coding, based on the study aims and the topics of the structured quantitative survey. The draft coding frame was discussed and refined with JH and CC prior to application to the dataset by LS and CC. LS and CC also identified patterns and themes of particular salience for participants and across the dataset using constant comparison techniques [33, 34]. Qualitative findings were integrated with quantitative findings (LS) in a narrative, using the ‘following a thread’ technique [35]. Qualitative data extracts are tagged with unique ID codes referring to participant role and case study trial number, using the following prefixes: TM trial manager, CI chief investigator, PC public contributor, and PPI Coord trial-specific PPI coordinator.

### PPI in this study

The study team included two public contributors (MD, KD) who were involved to ensure that the research was relevant to, and informed by the perspectives of, patients and members of the public. The two contributors were chosen because of their long-standing interest and involvement in PPI in trials. They provided feedback on the study design and topic guides, helped interpret the results and commented on the draft manuscript, helping in particular to ensure clarity and consistency.

## Results

26 TMs or CIs of current or recent trials were invited to participate in the survey; twenty-one TMs completed it; mean years of trial management experience 6.7 (standard deviation (SD) 4.88; range 0–15).

To achieve the target of eight case study trials, we approached nine TMs; one did not respond to the invitation. Across the eight case study trials, nineteen interviews were conducted, with public contributors (*n*=8), TMs (*n*=5), CIs (*n*=3), PPI coordinators (*n*=2) and a researcher (Table 1). No one who was approached for an interview declined. Details of the eight case study trials are given in Table 2.

**Table 1:** Interview participants across case study trials

Case study trial	Interview participants					Total
	Public contributor	Trial manager	Chief investigator	PPI coordinator	Researcher	
#1	2	1	1	0	0	4
#2	1	1	0	1	0	3
#3^a^	0	0	1	0	0	1
#4	1	1	0	1	0	3
#5	1	1	0	0	0	2
#6	3	0	0	0	1	4
#7^b^	0	1	0	0	0	1
#8	0	0	1	0	0	1
**Total**	**8**	**5**	**3**	**2**	**1**	**19**

^a^PPI members were children so none were interviewed for the study

^b^Could not interview PPI members as part of a panel working on several studies; the panel coordinator did not permit contact

**Table 2:** Characteristics of case study trials

Setting (population)	Design	Intervention	Summary of PPI	Stages of trial involving PPI
Community health (adults)	Individually randomised, two-arm pilot RCT	Counselling and psychotherapy	Primary role of PPI was to ensure study met the needs of target population. x2 PPI members contributed a few times during set up. x1 PPI member on TSC	Grant application Planning Ongoing oversight (TSC)
Primary care (adults)	Cluster-randomised two-arm RCT	Organisational, behavioural	Role of PPI to put pressure on the trial team, emphasising participants’ contribution/involvement and ensuring patient-centredness. (PPI perspective) Role of PPI to ensure research will benefit patients and that what we are doing in trial meets their needs/is important to them. (TM perspective) No PPI members on TMG. x2 PPI members in TSC and large PPI group of 10 members who meet every 3–4 months (including those on TSC). 2 additional members dropped out during the trial.	Planning Ongoing oversight (TSC) Implementation
Educational setting (adolescents)	Two-arm cluster RCT (feasibility and definitive)	Public health, behavioural	Same PPI group used throughout. x5 PPI members met once a year	Planning Implementation
Primary care (children)	Individually randomised, three-arm RCT	Pharmacological	Purpose of PPI is to ensure trial processes are acceptable to target patient group. x10 PPI members, ad hoc meetings every 2–4 months (same members throughout) x1 PPI member of TSC	Development/planning phase Ongoing trial oversight
Secondary care (adults)	Two-arm RCT	Surgical	Surgeons recruited PPI members (small number). 2–4 patients were asked their views during application stage. Different PPI people then became members of the TSC. But also looked at trial documentation outside of the TSC. x2 PPI members met every 5–7 months. 1x PPI member of TSC.	Planning/grant application Ongoing oversight
Community health (adults)	Two-arm RCT	Behavioural	Purpose of PPI to ensure taking account of the patient voice. x20 PPI members. Met every 5–7 months. PPI members included in TMG and TSC	Planning Ongoing oversight
Secondary care (adults)	Two-arm multi-centre RCT	Surgical	Consulted larger ‘general’ patient group on study design during application stage. Then a PPI group which more relevant condition experience was established specifically for study. The same group continued to support the study throughout its duration.	Planning/grant application Planned to use for dissemination once at that stage.
Primary care (adults)	Pilot Two-arm RCT	Behavioural	Met with different patient groups and forum to inform grant application, study approach and documentation. Specific numbers not indicated, however one group x 10 people.	Planning/grant application

Findings are presented in seven themes: the extent and organisation of PPI within trials, PPI purpose and changes due to PPI, training of public contributors and trial staff, payment and reimbursement**,** challenges and barriers to successful PPI, facilitators and drivers of successful PPI and the role of CTUs.

### Extent and organisation of PPI within trials

In survey responses, 15 trials (71%) had one or more public contributors on the trial team (either on Trial Steering Committee (TSC) (*n*=8), Trial Management Group (TMG) (*n*=6) or both TSC and TMG (*n*=1)). Five TMs reported accessing the views of members of the public in another way; for example via consultation of a separate RCT PPI group, having public contributors on the RCT advisory group or consultation with local patient groups. One trial manager was unaware of any PPI in their trial.

The 15 trials that included public contributors on the TMG or TSC recruited 1–20 public contributors (mean 6.4, 1 missing), but in practice 0–15 contributors (mean 4.8, 2 missing) were regularly involved in providing PPI input during the trial. Ten TM survey respondents reported that they had asked public contributors for feedback regarding their experiences of contributing to the RCT (data integrated below).

### PPI purpose and changes due to PPI

In the survey, TMs reported that a wide range of tasks was undertaken by public contributors (Table 3); the most common were the review of participant-facing materials and other study documents and advising on recruitment/retention strategies. Some trials had collaborated with public contributors in coding qualitative data and disseminating findings. Thirty-one different types of changes were reported due to PPI, most commonly related to trial documentation (*n*=18, 58.1%) and trial design (*n*=10, 32.3%). Four TMs reported no changes had yet been made because of PPI.

**Table 3:** Tasks undertaken by PPI representatives (survey and case studies)

Task	Reported by TMs surveyed (***n***=21) ***N*** (%)	Case study trial							
		#1	#2	#3	#4	#5	#6	#7^**a**^	#8^**a**^
Review of participant-facing materials (e.g. participant information sheets)	14 (66.7)	Yes	Yes	Yes	Yes	Yes	Yes	Yes	Yes
Review of other study documentation (e.g. protocol, reports)	11 (52.4)	Yes		Yes					
Advising on recruitment and retention	11 (52.4)	Yes		Yes	Yes	Yes	Yes		Yes
Involvement in protocol development	10 (47.6)	Yes			Yes	Yes	Yes	Yes	Yes
Trial design	10 (47.6)	Yes	Yes		Yes	Yes			
Member of Trial Steering Committee (TSC)	9 (42.9)	Yes	Yes		Yes	Yes	Yes		
Dissemination	8 (38.1)						Yes		
Member of Trial Management Group (TMG)	6 (28.6)	Yes				Yes	Yes		
Agreeing study logistics	6 (28.6)			Yes					
Funding application development	6 (28.6)	Yes		Yes	Yes	Yes			

^a^Survey question not answered, data from interviews

Documents changed as a result of PPI input included invitation letters and other recruitment materials, information sheets, questionnaires and other data collection instruments, intervention materials, newsletters, website content and dissemination materials. Aspects, some of which were major, of trial design changed as a result of PPI input included whether to adopt a two- or three-arm design; inclusion/exclusion criteria; intervention design; data collection methods including topic guides, outcome measures and selection of feasible time points; recruitment and consent processes; and ways to reduce participant burden and increase retention.

Interview participants described revising the content of interventions as well as participant-facing documents on the basis of public contributors’ input. A CI described how public contributors helped determine the name and clothing of the ‘peer supporters’ delivering the intervention:

We asked [public contributors] whether – what should peer supporters be called? Um should they be ‘peer supporters’? Should they be ‘mentors’? Should they be something else? We asked them what the peer supporters should wear at the training. CI 1

Trial teams could be constrained when trying to implement public contributors’ feedback by a lack of flexibility in trial design; for example the need to the use of validated outcome measures.

They didn’t really change the design in terms of what our outcome measures were… because we were kind of stuck with using questionnaires that were already validated, even though they [public contributors] didn’t like them and sometime it was quite frustrating that we couldn’t really make as many changes as we would have liked. TM 5

In the survey, fourteen TMs reported that changes made as a result of PPI were fed back to public contributors; one reported that they were not (four ‘not applicable’ as no changes had been made). In qualitative interviews, respondents acknowledged that formal feedback was not always provided regarding whether and how public input had been used in the trial. Public contributors wanted and valued this feedback:

It would be nice if there was more formal feedback given…like whether or not they sort of had written list of changes they had made from the feedback given and that would definitely help make one feel more involved in the process. PC 12

The main positive was seeing that… specific parts of my feedback had been taken into consideration between the first and second revision of the material. PC 2

### Training of public contributors and trial staff

Only five survey respondents (24%) reported that training was provided for public contributors; 12 (57%) reported no training and 3 (14%) did not know (missing *n*=1). Training usually focussed on the role, expectations from both sides, and the specific trial. One TM reported that public contributors received a day of study-specific internal training and were also offered external training on PPI from a local provider. TMs reported that public contributors valued the training when provided.

Two of the eight public contributors interviewed had received formal generic ‘research methods’ training through an external training provider, but both felt it would have been more useful if had been specific to the trial they were working on:

It struck me as saying ‘we’ll give you some educational input’, but I was frustrated because I thought well, let’s look at that in terms of discussing what these research methods etc. what implications does it have for the study. PC 1

The remaining six public contributors had not received any form of formal training, and felt training would have been useful for informing them what to expect in the role and what was expected from them.

Might benefit from knowing, well what am I doing here now? How do I actually act? What am I supposed to do? PC 14

Few academic staff, including those staff with a role coordinating PPI, had received formal PPI training, but had rather learned about PPI ‘on the job’:

I’m kind of self-teaching as I go along. So, I read a huge amount… I didn’t have any formal training on how-to-do good PPI. PPI Coord 2

### Payment and reimbursement

Twelve TMs surveyed reported that public contributors were paid for their time, four said the payment was not offered and three did not know (not applicable *n*=1, missing *n*=1). Payments varied widely, ranging from £10–£50/h depending on the trial and task. Three trials are paid in vouchers, the remainder by bank transfer or cash.

In interviews, problems with payment via the university system were reported:

Right at the start I had conversations with people in Finance [who confirmed] they didn’t need to do it on an expenses claim, but that position seems to have changed… it adds to the burden of the group that they have to then complete forms and then they get taxed and they don’t get that back until the end of the tax year. PPI Coord 1

Two of the public contributors indicated that sometimes the payment/vouchers offered appeared tokenistic and did not appropriately account for the time and effort required from representatives.

I thought that was a bit, £10 is a bit derisory. PC 1

I have had a few vouchers but certainly not as many as the amount of time I have spent doing it. PC 3

### Challenges and barriers to successful PPI

Survey respondents and interviewees discussed a range of challenges to implementing successful PPI, related to recruitment, representativeness, availability; managing group dynamics; maintaining professional boundaries; attitudes to PPI; lack of continuity of trial staff; and the academic environment.

#### Recruitment

In the survey, none of the TMs reported the use of a formal process to recruit public contributors to the TMG/TSC (informal process *n*=14, not known *n*=1). Most trials relied on local or national patient groups and contacts within the university or local health services to recruit public contributors. The most common reasons for selecting public contributors were expertise/experience in the subject area (*n*=13), previous experience of PPI (*n*=6) and being known to members of the TMG (*n*=4; respondents could select more than one reason). An interview participant commented:

When the trial actually got up and running… then two patient [representatives] were found. One was found because our chief investigator had just operated on him… and the other person… was doing some work for another trial. TM 3

In interviews, trial staff reported that it was difficult to recruit appropriate public contributors due to a lack of knowledge of how to access people and limited funding. Interviewees described how most public contributors were highly educated and from the trial lead site location and often where the CI was based, which could be problematic for a national trial:

A lot of our PPI group are university-based… they’re aware of how studies are run, and it’s been difficult to engage people beyond that really… they will generally be highly educated people… I don’t really know how to reach [other] people. TM 4

The only limitation is that this is a big multi-centre trial and we have only got 5 people from [name of city]… it would be nice to have a wider perspective from the other sites but it’s very difficult to coordinate public involvement across the country unless you have a lot of money. TM 5

#### Background

Related to this, public contributors on the trials were often ‘professional PPI members’ with significant experience of the PPI role and at times a different agenda to the study population:

The whole point about PPI is that they are meant to representative of the target study population. However, we have found that our PPI group are more active, intellectual and often have their own agendas. Therefore, you end up with 'professional PPI' members that get involved in lots of different studies but push a slightly different agenda to the study populations that they are meant to be representing. TM (survey response)

Of the eight public contributors interviewed for the study, four had a professional background in academia and research.

#### Availability

The fact that trial meetings usually took place generally during work/school hours meant it was difficult to find public contributors who would be available:

We really struggled to have people who would be able to become free – I think that was due to timing. The time of the school day and [when] our meetings are scheduled... CI 1

In the survey, TMs discussed public contributors’ frustration at changing meeting dates.

#### Managing group dynamics

In interviews, TMs described how managing complex group dynamics to try to ensure multiple perspectives were captured could be challenging:

You always have some outspoken members of the group and everyone else kind of tends to hide behind them and it almost becomes a one-on-one consultation, I think that’s where very good group management [is needed] – there is a certain amount of skill involved in trying to keep people involved… trying to get them actively interested in the area. TM 5

From the public contributor perspective, the group dynamics of research professionals and clinicians who were familiar with each other and with the study could be challenging and lead to a reluctance to engage:

There’s always with these groups a sense of knowledge shared between them which makes them move through parts of the agenda with the kind of ease and a flow which could leave PPI members behind… there are times when you feel this flow is difficult to interrupt. PC 15

#### Maintaining professional boundaries

Another reported challenge was the difficulty of managing and maintaining professional boundaries between public contributors and trial staff:

It also became slightly complicated in that [the PPI contributor] was then looking at me for help with the research project that she’s involved in and so yeah it was quite difficult to think about, you know, because she needs to be an independent member and then if I was going to be involved in some work with her that was going to make her non-independent, so I had to clarify those boundaries which felt difficult. CI 2

[Personal and professional] Boundaries are a real issue… [The PPI contributor] is quite demanding timewise… so thinking about lessons learned – that idea of not just sort of clarifying expectations but also setting boundaries or agreeing boundaries in some way if there’s an issue about that; and I have had some – a few very direct conversations with [him/her] where I’ve… absolutely laid down the boundaries because it’s just getting unmanageable. TM 1

#### Attitudes to PPI

In the survey, TMs reported positive feedback from public contributors when CIs joined in with PPI meetings, listened to what public contributors had to say and were made to feel like a valued and integral part of the team, for example by being included in publications. While many public contributors did felt supported in this way, some interview respondents reported that public contributors were not always valued by all trial team members, with some researchers questioning their usefulness:

There was one member of the team who doesn’t welcome anybody…I think sometimes there is a bit of ‘Oh, we have got to jump through the PPI hoop for the trial’… I think it’s becoming better respected and better valued, but again I think it’s that concern from some, of how representative and truly informative it is. PC 13

#### Lack of continuity of trial staff

Another challenge highlighted was how ways in which staff were employed on trials caused a lack of continuity:

Obviously, it is hugely important to get PPI better embedded in trials- I think it is difficult to do this effectively with the changing workforce associated with trials, and the fact that researchers are often employed after the study has been designed, which causes a lack of continuity. TM (survey)

#### Academic environment

Finally, interview respondents discussed how the academic environment and format of TMG and TSC meetings could be difficult, with some public contributors feeling nervous, apprehensive or lacking confidence to speak. This was also reported to have implications for representativeness:

I think they would probably be quite nervous… I think there’s a barrier there. So I think you’d need to be quite a confident, self-assured um teenager to sit and go to – firstly come to University, um sit in a – in a room with a bunch of um of adults and – and think that your opinion would hold water. CI 1

It could be quite intimidating to sit in front of these guys and raise questions which may seem absolutely obvious for everyone around the room and yet without somewhat embarrassed about the and therefore not saying anything. PC 6

Similarly, survey respondents mentioned public contributors’ negative feedback regarding the use of technical research-related jargon.

### Facilitators and drivers of successful PPI

Respondents highlighted many ways in which successful PPI could be facilitated, in three main areas: planning, sharing ownership and communication. Funders’ role in driving good PPI was also discussed.

#### Planning

Considering and planning PPI from the start of a trial (including when developing the grant application and protocol) were reported to be key to successful PPI. Good planning meant that relationships were established early on, which could be helpful in responding to difficulties as the trial progressed:

When you – you do a bit more research you realise that actually things don’t work in the way that you planned or recruitment efforts – efforts don’t work or people don’t respond to the things that you think they will do um and so really kind of engaging people from the beginning is pretty wise. CI 1

A clear plan which outlined the timing, remit and purpose of the PPI also ensured it was properly prioritised:

I think making it a priority from the beginning and having a particular aim for the PPI, saying, you know, it’s not piecemeal, we are not just gonna [say], ‘We’ll talk to patients about what they think of the whatever’; I think saying, you know, we will specifically talk with [PPI] at this point in the grant and ask them about these things so that we can do something with the results… that gives it purpose, because otherwise I think if it’s vague, then it gets lost amongst other priorities. CI 1

Part of planning successful PPI was reported to include thinking about and asking key questions of the public contributors:

We’re not asking questions for the sake of it. We’re not saying, ‘what do you think of this, isn’t it nice?’ We’re asking questions because we need to know the answer… So you ask pertinent questions, you ask questions that they are actually well placed to answer. And then you listen to what they have to say. PPI Coord2

#### Sharing ownership

Establishing an ongoing relationship with public contributors and an iterative consultation process was reported to help facilitate continued engagement:

We have had, say, four or five patients… who have been engaged from the very beginning and I think it’s quite an achievement that they stayed on board with the project. And part of it is because they have got someone who can coordinate regular meetings and give them updates with how things have [been] running and get various people who talk to them and keep them interested generally in the idea… so we have done our bit to keep it interesting for them. TM 5

Public contributors enjoyed being involved and valued contributing to the research, highlighting that inclusion and openness to their suggestions were key:

I think that’s something which research groups need to be aware of. If they are going to use what a PPI member can offer then I think they probably need to give some thought to that, the ways in which they can sort of exclude people… At all the meetings they’ve always made a point of saying, ‘Well, is there anything you would like to add to that?’ PC 14

#### Communication

Survey respondents and interviewees thought it was important to ensure public contributors understood what was expected of both parties and what was involved in the research process and their role in it. This proactive, clear communication from academic members of the trial team was needed early on so that public contributors could make informed decisions about whether to take on the role and what it would entail:

Be very clear about the parameters of the PPI involvement and make sure that people really understand what it is that you want from them at the outset, so that they can make a really informed decision, just as you would when someone’s taking part in the main research. TM 2

I think understanding the research process is useful… what are we trying to do? As researchers, what is our aim? And what’s our position of – and what’s our view of the world, why are we doing this? Why do we think it’s important? So them [PPI] understanding that but also mainly understanding their role in it. CI 1

In the survey, TMs reported positive feedback from public contributors when varied contact methods (email, telephone, face-to-face) were offered.

#### Funders’ role

Funders’ increasing recognition of the importance of PPI was reported to be a driver for effective PPI:

NIHR take it seriously… it is very clear from their website and all the materials you read about INVOLVE and so forth that it – it’s taken seriously by the funder so you know, if we want this – the funder to think we’ve been doing a good job, then we really need to – to tick that box as well. CI 1

### Role of CTUs

TMs and CIs who were surveyed and interviewed reported that a CTU could support PPI in trials via assistance with recruitment of public contributors, sign-posting and coordination.

#### Recruitment

Respondents described how CTUs could assist with the recruitment of public contributors, either via providing a list of people interested in providing PPI, offering access to a panel of trained public contributors at different life stages, or sign-posting to specific patient groups:

Having access to a pool of interested parties where you can pull people who are most representative of the patient groups or whatever study you are working on. TM 1

If the [CTU] had a PPI panel… that would be useful… an engagement panel of a kind of citizens panel… like of life course stages. CI 1

#### Signposting and coordination

Respondents described the value of a CTU signposting to existing training, and up-to-date guidelines, examples and resources related to PPI (e.g. detailing the value of PPI at different trial stages or how to integrate PPI from the start):

A central repository of all those sorts of resources might be useful. Someone, somewhere, where people can go to for any specific advice. TM 1

A CI explained how access to better resources could help:

Having a website of resources for researchers would be helpful. I think that could only be a good thing for the success rate of the projects… in terms of getting funding but then also the success of the projects themselves... I mean, anything we can do to get a competitive advantage. CI 1

Extending the idea of signposting, some respondents described the potential utility of CTUs providing a dedicated PPI coordinator to advise on PPI and support researchers working on trials with their PPI activities.

Having somebody who is scanning the horizons for what’s new and what’s the new thinking, where – where – you know, are there new – is there new guidance from different boards, so has the MRC [Medical Research Council] got guidance on PPI, which is different to the NIHR? CI 1

## Discussion

In this study of patient and public involvement at one UK clinical trials unit, we found that although PPI was included in 71% of trials surveyed, the extent of involvement was variable and managed inconsistently, and significant challenges to successful PPI were reported. Public contributors played a valuable role, particularly in ensuring the appropriateness and acceptability of trial design and participant-facing documentation. However, public contributors were recruited informally and on an ad hoc basis, and the provision of training was variable. Public contributors valued receiving training, feeling integral to the trial team and receiving formal feedback on changes made because of their input. However, public contributors were not valued by all trial staff; one described how they felt PPI was seen by some researchers as a ‘hoop’ that had to be jumped through. Payment varied across trials and tasks; while payment according to the task is in line with NIHR guidance [36], some trials appeared to be underpaying public contributors. Reported challenges in implementing successful PPI included difficulties recruiting appropriate PPI representatives, “professional” PPI members reportedly pushing their own agenda, and maintaining professional boundaries.

Kearney et al. [20] found that a top priority for PPI methodological research was developing strong and productive relationships between researchers and PPI contributors. Our research found that such relationships are essential to the successful implementation of PPI and depend on researchers planning PPI well, sharing ownership and communicating proactively and clearly. Supporting the findings of the EPIC study of HTA-funded trials [37], we found that PPI needs to be considered and organised early in the trial process, with roles and expectations made clear on both sides. In addition, our findings highlight how establishing ongoing relationships with public contributors and an iterative consultation process helped them feel included and enabled more effective PPI.

We found variation in PPI models across trials: including public contributors as members of trial oversight committees, consultation of a separate trial PPI group, having public contributors on the trial advisory group or consultation with local patient groups. Case study research by the Medical Research Council CTU at University College London lists similarly varied models [38]. The suitability of a specific model of PPI will depend on who the trial is aiming to involve or recruit, and it is crucial that both the model and method of PPI are appropriate to the population group and inclusive. A study of PPI in surgical trials, for example found that a two-tier model of PPI, in which a small number of public contributors are closely involved with the trial team and linked to a larger group of patients, was beneficial because it resulted in a better representation and patient engagement than the involvement of one or two PPI contributors alone [39]. This was not a model reported by the trials in this study; future research could explore the feasibility and value of such a model across diverse trials. Respondents in our study felt that CTUs could help support PPI implementation in trials through supporting recruitment (e.g. providing access to PPI panels), funding a PPI coordinator post and/or signposting to appropriate resources and training—these suggestions informed the development of PPI support at the CTU (described below).

This study provides a broader perspective on PPI in trials which complements ethnographic research on PPI specifically within trial oversight [23], where counter-productive views were reported about public contributors amongst some trial committee members, as in this study. Similarly, both studies identified concerns that ‘professionalised’ (and, in this study, academic) PPI contributors may not represent the ‘average’ member of the public. It is possible that professionalised contributors might be selected by researchers for PPI roles as they fit more easily into academic processes and require less adaptation by research teams [21, 40]. However, the charge of ‘professionalism’ should not be used to undermine the contribution of a PPI team member. The process of PPI is inherently relational, often involving negotiating multiple identities, power imbalances and ambiguitie s[41, 42]. Furthermore, the involvement of more experienced public contributors or those with an academic background does not necessarily disadvantage a trial: the suitability of the PPI member (and the advantage or disadvantage of more research experience or an academic background) depends on the specific task and trial [43]. Arguably, where a public contributor is able to bring both relevant personal experience and an informed professional perspective this could be of benefit to the trial. These nuances suggest that the recruitment and selection of PPI contributors should be transparent and carefully considered, taking into account the range of perspectives needed to inform the research and providing a mix of skills and experience [44].

### Strengths and limitations

A strength of our study was the use of a sequential mixed-methods design in which survey findings informed case study sampling and in-depth interview data were triangulated with survey data. This provided a more in-depth understanding of the implementation of PPI in trials and enhances the richness and validity of our findings. We recruited a diverse range of people involved in implementing PPI in trials, including eight PPI representatives as well as trial managers, PPI coordinators and CIs, providing multiple stakeholder perspectives. The study was conducted at a single CTU in the UK, and other CTUs may be organised differently (for example, employing a pool of TMs to run trials rather than supporting CI-led trials). All the trials were all conducted in the UK. Findings might not be transferrable to CTUs outside the UK or which focus on international trials. Although we included only trials which ended in the 2 years prior to data collection, we relied on retrospective reports regarding past trials hence responses are subject to recall bias. Data collection occurred 5 years prior to publication, hence findings may not be transferrable to current trials; however, findings from an earlier stage of PPI development nonetheless provide useful evidence to inform current practice. The study was cross-sectional, hence we were not able to capture any change in views and practices over time nor whether implementing specific strategies made a difference to trials or PPI experience.

### Implications for practice and recommendations

Findings from this study informed the development of PPI support at the CTU, which has changed considerably in the last 5 years. In particular, from 2017, the CTU appointed a PPI to lead signpost CIs and TMs to PPI training, guidance and recruitment resources, including a database of local and national organisations that support PPI. Since 2019, there has also been a PPI working group at the CTU that focusses on disseminating good practice in PPI by devising local policies and procedures, developing support materials for PPI contributors and signposting national resources and existing colleagues in the university. The CTU has also worked with NIHR Evaluation, Trials and Studies Coordinating Centre (NETSCC) to help devise national guidance for PPI in trial oversight and has run a national workshop for training TSC Chairs with a session on PPI co-delivered by a public contributor. Many of these initiatives relate directly to the recommendations of study participants. However, the CTU has not introduced a Standard Operating Procedure (SOP) for PPI as the team has found the coordinator and signposting to be effective and an SOP could potentially be unnecessarily prescriptive.

Through these initiatives, and in parallel with other CTUs, in recent years, the CTU has worked to ensure PPI in the design and conduct of portfolio trials. PPI is actively used in the design phase of most definitive trials, with public contributors usually included as co-applicants. A specific study team member, often the TM, acts as a liaison and mentor for trial PPI members. All TSCs and most TMGs now include PPI members. There are often briefing or debriefing meetings before TMG or TSCs meetings to enhance PPI contributions. The CTU ensures meetings are held at appropriate times/venues with additional facilitation for virtual meetings, such as organising a practice meeting. PPI members are compensated appropriately for their contributions according to national guidance. Trials either have several individual PPI contributors or an Advisory Group comprised of several individuals; our experience is that the latter can work well for a longer-running trial or a trial with an older or unwell population. Participant-focused newsletters are used to maintain engagement and lay summaries of the main results have been prepared for several trials. These summaries, which for some trials also include videos and infographics, are made available via the trial website.

More generally, study findings contribute to our understanding of PPI in trials and are relevant to guidance and training in this area [45]. This study includes phase III trials across a range of settings, including primary care and public health, extending the findings from previous investigations of PPI in trials in surgery [39], health care [46], cancer and HIV [38], secondary care [23] and a cohort of secondary or tertiary care and one community-based trial funded by the NIHR HTA programme between 2006 and 2010 [19]. Our findings support and add to the ‘lessons learnt’ outlined by South et al. [38] and the recommendations made by Coulman et al. [23] on implementing successful PPI in trials. In particular, our findings support a focus on planning an integrated approach to PPI at an early stage of a trial; recruiting diverse and appropriately skilled and experienced public contributors, according to the needs of the trial; and ensuring the process of PPI sufficiently enables and supports successful collaboration (e.g. through training and preparation of both researchers and public contributors, and adaptation of formal academic meetings to enable co-ownership). Bagley et al. have developed resources for providing clear information about trial oversight to public members, including their role [45]. Our research flags inconsistencies in payment practices, suggesting the NIHR Centre for Engagement and Dissemination guidelines [36] are not consistently followed but also that university payment systems can impede prompt payment. We recommend that researchers use the NIHR Centre for Engagement and Dissemination payment structure [36], but also work with university finance teams to ensure payment to public contributors is straight-forward and timely. Our findings also add detail regarding how a CTU can best support PPI in trials, through assistance with in recruitment, sign-posting to current resources, guidelines and training and helping with coordination of PPI, for example through funding a specific coordinator post within the CTU. As described, based on study findings the CTU has developed and implemented several initiatives to help support good PPI in trials. CTUs also play an important role in auditing PPI in the trials it supports and ensuring PPI is adequately evaluated: less than half of the portfolio trials we surveyed had evaluated their PPI via feedback from public contributors, and this is an area which CTUs can help improve.

## Conclusions

Public contributors play a highly valuable role in clinical trials, helping to ensure design and conduct are appropriate, acceptable, relevant and high quality. However, the implementation of PPI in trials is variable, with inconsistencies in the training and payment of public contributors. Barriers to successful PPI included recruitment challenges, managing group dynamics, maintaining professional boundaries, negative attitudes to PPI amongst some researchers, a lack of continuity of trial staff and aspects of the academic environment. Conversely, facilitators of successful PPI included early and explicit planning of PPI (starting during trial development), sharing ownership with public contributors, ensuring they feel valued, establishing an iterative consultation process and communicating proactively and clearly about expectations and roles. Funders help drive successful PPI through their funding requirements, and CTUs have an important role to play in supporting recruitment, signposting and coordinating PPI.

## Supplementary Information

**Additional file 1.** Online survey for Trial Managers/PIs

**Additional file 2.** Topic guide for interviews with trial management group members

**Additional file 3.** Topic guide for interviews with PPI representatives

## Data Availability

The survey questions and interview topic guides are available in supplementary materials. Data is available on reasonable request.

## Abbreviations

CI: Chief Investigator
CTU: Clinical Trials Unit
NETSCC: NIHR Evaluation, Trials and Studies Coordinating Centre
NIHR: National Institute for Health Research
PC: Public contributor
PPI: Patient and Public Involvement
PPI coord: PPI coordinator
RCT: Randomised controlled trial
SOP: Standard Operating Procedure
TM: Trial manager
TMG: Trial Management Group
TSC: Trial Steering Committee
UKCRC: UK Clinical Research Collaboration
