# Supplementary material for: Patient and public involvement in randomised clinical trials: a mixed-methods study of a clinical trials unit to identify good practice, barriers and facilitators
Source: Trials. 2021 Oct 23;22:735. doi: 10.1186/s13063-021-05701-y (PMC8542312; doi:10.1186/s13063-021-05701-y)
Supplement: Supplementary file 1 — Additional file 1. Online survey for Trial Managers/PIs [file 13063_2021_5701_MOESM1_ESM.docx]

**Additional file 1: Online survey for Trial Managers/PIs**

Introduction:

You are going to be asked some questions about yourself, the trial(s) you have been involved in, and your opinions about patient and public involvement in trials. All your responses will be recorded anonymously – we will not record your name or any details that that could identify you. Storage of all data will comply with the Data Protection Act 1998 and University of Bristol’s data protection policies and stored securely on University computer systems. If you are happy to continue please touch the start button below.

What is the name of the most recent BRTC-portfolio trial you worked on or are currently working on?

Are you male or female?

How many years have you been working in trial management?

How old are you?

*The next questions relate to Patient and Public Involvement (PPI) in the trial named above.*

How were PPI representatives accessed?

• Already existing patient group – please state

• Via a charity – please state:

• Via website – please state

• Via previous work in SSCM project

• Other – please state

Was it a formal recruitment and appointment process or informal?

How many PPI representatives were recruited?

How many PPI representatives were actually involved?

What was PPI representatives’ background? (Open question)

Why were PPI representatives selected? (Tick as many as apply):

• Expertise in subject area

• Previous experience of PPI

• Familiar to members of Trial Management Group

• Other – please state:

Tasks undertaken by PPI representatives (tick as many as apply):

• trial design

• funding application development

• involvement in protocol development

• document review

• agreeing study logistics

• advising on recruitment and retention

• members of Trial Management Group

• Member of Trial Steering Committee

• dissemination

PPI commitment

• How often did PPI representatives attend face-to-face meetings?

• How long were the meetings?

• Were PPI representatives asked to complete tasks via email?

What changes were made as a result of PPI?

• Changes to trial design– please state:

• Changes to documentation– please state:

• Other changes – please state:

• No changes were/have been made

Where changes made communicated to PPI?

• Yes

• No

• Not relevant

Were PPI representatives paid for their work?

• Yes – rate: approximate payment per hour/day:

• No, only travel expenses

Was any training provided for PPI representatives? (Y/N)

Was any written information given to PPI representatives to recruit them/define their role? (Y/N)

In your opinion, what support is required from the CTU to support PPI within trials? (Open question)
