# Supplementary material for: Patient and public involvement in randomised clinical trials: a mixed-methods study of a clinical trials unit to identify good practice, barriers and facilitators
Source: Trials. 2021 Oct 23;22:735. doi: 10.1186/s13063-021-05701-y (PMC8542312; doi:10.1186/s13063-021-05701-y)
Supplement: Supplementary file 2 — Additional file 2. Topic guide for interviews with trial management group members [file 13063_2021_5701_MOESM2_ESM.docx]

**Additional file 2: Topic guide for interviews with trial management group members**

What is your opinion about patient and public involvement in research?

Brief description of PPI involvement in the project

Role of PPI members and tasks conducted

Were the roles of PPI members adequately fulfilled?

How did the tasks go – were any changes made as a result?

What went well?

Probes:

What enabled/facilitated successful PPI?

What did not go so well?

Probes:

Were there any barriers to successfully working with PPI representatives?

Is there anything you would do differently next time?

What was the value (if any) of PPI involvement in this study?

How did you manage working relationships with PPI members?

What support (if any) is required from BRTC to support PPI within trials?
