# Supplementary material for: Patient and public involvement in randomised clinical trials: a mixed-methods study of a clinical trials unit to identify good practice, barriers and facilitators
Source: Trials. 2021 Oct 23;22:735. doi: 10.1186/s13063-021-05701-y (PMC8542312; doi:10.1186/s13063-021-05701-y)
Supplement: Supplementary file 3 — Additional file 3. Topic guide for interviews with PPI representatives [file 13063_2021_5701_MOESM3_ESM.docx]

**Additional file 3: Topic guide for interviews with PPI representatives**

Brief description of PPI involvement in the project

How were you recruited to join the trial?

Why did you take on the role?

Did you have any previous experience of working in PPI?

What were your roles in the trial?

What went well?

Probes:

What enabled/facilitated successful PPI?

What did not go so well?

Probes:

Were there any barriers to successfully working on the study?

Is there anything you would do differently next time?

Is there anything you would suggest the trial team does differently next time?

What was the value of PPI involvement in this study?

Did you feel PPI was appreciated/valued by the trial team?

Were changes made as a result of PPI fed back to the PPI members?

Were you provided with any training?

Would you work as a PPI representative again?
